# Effect of a Patient Decision Aid on Lung Cancer Screening Decision-Making by Persons Who Smoke: A Randomized Clinical Trial

**DOI:** 10.1001/jamanetworkopen.2019.20362

**Published:** 2020-01-31

**Authors:** Robert J. Volk, Lisa M. Lowenstein, Viola B. Leal, Kamisha H. Escoto, Scott B. Cantor, Reginald F. Munden, Vance A. Rabius, Linda Bailey, Paul M. Cinciripini, Heather Lin, Ashley J. Housten, Pamela Graef Luckett, Angelina Esparza, Myrna C. Godoy, Therese B. Bevers

**Affiliations:** 1 Department of Health Services Research, The University of Texas MD Anderson Cancer Center, Houston; 2 Department of Health Disparities Research, The University of Texas MD Anderson Cancer Center, Houston; 3 Department of Radiology, Wake Forest School of Medicine, Winston-Salem, North Carolina; 4 Department of Behavioral Science, The University of Texas MD Anderson Cancer Center, Houston; 5 North American Quitline Consortium, Phoenix, Arizona; 6 Department of Biostatistics, The University of Texas MD Anderson Cancer Center, Houston; 7 Information & Quality Healthcare Inc, Ridgeland, Mississippi; 8 Houston Department for Health and Human Services, Houston, Texas; 9 Department of Diagnostic Radiology, The University of Texas MD Anderson Cancer Center, Houston; 10 Department of Clinical Cancer Prevention, The University of Texas MD Anderson Cancer Center, Houston

## Abstract

**Importance:**

Lung cancer screening with low-dose computed tomography lowers lung cancer mortality but has potential harms. Current guidelines support patients receiving information about the benefits and harms of lung cancer screening during decision-making.

**Objective:**

To examine the effect of a patient decision aid (PDA) about lung cancer screening compared with a standard educational material (EDU) on decision-making outcomes among smokers.

**Design, Setting, and Participants:**

This randomized clinical trial was conducted using 13 state tobacco quitlines. Current and recent tobacco quitline clients who met age and smoking history eligibility for lung cancer screening were enrolled from March 30, 2015, to September 12, 2016, and followed up for 6 months until May 5, 2017. Data analysis was conducted between May 5, 2017, and September 30, 2018.

**Interventions:**

Participants were randomized to the PDA video *Lung Cancer Screening: Is It Right for Me?* (n = 259) or to EDU (n = 257).

**Main Outcomes and Measures:**

The primary outcomes were preparation for decision-making and decisional conflict measured at 1 week. Secondary outcomes included knowledge, intentions, and completion of screening within 6 months of receiving the intervention measured by patient report.

**Results:**

Of 516 quit line clients enrolled, 370 (71.7%) were younger than 65 years, 320 (62.0%) were female, 138 (26.7%) identified as black, 47 (9.1%) did not have health insurance, and 226 (43.8%) had a high school or lower educational level. Of participants using the PDA, 153 of 227 (67.4%) were well prepared to make a screening decision compared with 108 of 224 participants (48.2%) using EDU (odds ratio [OR], 2.31; 95% CI, 1.56-3.44; *P* < .001). Feeling informed about their screening choice was reported by 117 of 234 participants (50.0%) using a PDA compared with 66 of 233 participants (28.3%) using EDU (OR, 2.56; 95% CI, 1.72-3.79; *P* < .001); 159 of 234 participants (68.0%) using a PDA compared with 110 of 232 (47.4%) participants using EDU reported being clear about their values related to the harms and benefits of screening (OR, 2.37; 95% CI, 1.60-3.51; *P* < .001). Participants using a PDA were more knowledgeable about lung cancer screening than participants using EDU at each follow-up assessment. Intentions to be screened and screening behaviors did not differ between groups.

**Conclusions and Relevance:**

In this study, a PDA delivered to clients of tobacco quit lines improved informed decision-making about lung cancer screening. Many smokers eligible for lung cancer screening can be reached through tobacco quit lines.

**Trial Registration:**

ClinicalTrials.gov identifier: NCT02286713

## Key Points

**Question:**

Does providing a lung cancer screening decision aid through tobacco quitlines improve informed decision-making about lung cancer screening among persons who smoke?

**Findings:**

In this randomized clinical trial of 516 smokers, use of a patient decision aid compared with standard educational information led to better preparedness to decide about screening, higher reports of feeling informed and clear about screening choices, and greater knowledge of screening benefits and harms.

**Meaning:**

The findings suggest that decision aids about lung cancer screening can reach large numbers of smokers who are eligible for screening through tobacco quitlines, can inform them about lung cancer screening, and can promote high-quality screening decisions.

## Teaser

This randomized clinical trial compares the effect of a patient decision aid on lung cancer screening vs standard educational information on decision-making outcomes among persons who smoke.

## Introduction

Lung cancer is the leading cause of death from cancer in the United States, and smoking is the most important risk factor for developing and dying of lung cancer.^1,2^ The National Lung Screening Trial^3^ found 20% fewer lung cancer deaths among current and former heavy smokers screened using low-dose computed tomography (LDCT) compared with those screened with standard chest radiography.^3^ However, screening rates nationally remain low.^4^ In addition, lung cancer screening with LDCT is not without risks, including radiation exposure from screening and diagnostic imaging and a high false-positive rate leading to subsequent testing, which also is associated with harms.^5,6^

Guidelines about lung cancer screening are consistent in emphasizing the importance of patients making an informed decision within the context of receiving smoking cessation services for people who continue to smoke.^7,8,9,10,11,12,13,14^ The Centers for Medicare & Medicaid Services (CMS) has financially covered lung cancer screening using LDCT since 2015, but the CMS guidelines require a patient counseling and shared decision-making visit using patient decision aids (PDAs) before screening referral.^15^ The requirement to use PDAs for CMS reimbursement of lung cancer screening is unprecedented. There is a need for PDAs to support informed decision-making about lung cancer screening using LDCT, yet few tools have been developed and none have been evaluated in comparative trials.^16^

We undertook a randomized clinical trial (see trial protocol in Supplement 1) of a previously developed PDA video for lung cancer screening^17,18^ to examine its effect on (1) smokers’ preparation for having a conversation with a health care clinician about lung cancer screening, (2) assuredness about a screening decision, (3) knowledge of lung cancer screening, (4) intentions to be screened, and (5) completion of screening. Our target population included persons seeking smoking cessation services through tobacco quit lines who met screening eligibility criteria based on age and smoking history. Tobacco quit lines were selected as the study setting because smoking cessation is an essential component of lung cancer screening programs,^19^ and tobacco quit lines provide services to many individuals at high risk of lung cancer.

## Methods

### Study Design

This randomized clinical trial tested the outcomes of a participant using a PDA about lung cancer screening on the decision to be screened for lung cancer. Participants (clients) were recruited through tobacco quit lines. Baseline, 1-week, 3-month, and 6-month follow-up assessments were conducted. Participants who could not be reached by telephone received the study questionnaires by mail. Recruitment lasted from March 30, 2015, to September 12, 2016, and follow-up assessments were completed by May 5, 2017. Participants were compensated $50 after the 1-week assessment and $25 at the 3- and 6-month follow-ups. The protocol for this study has been published elsewhere.^20^ The institutional review board of The University of Texas MD Anderson Cancer Center, Houston, Texas, approved the study before data collection (eTable 1 in Supplement 2). Participants provided oral informed consent following a presentation of the study by a research coordinator. This study followed the Consolidated Standards of Reporting Trials (CONSORT) reporting guideline.

### Setting and Participants

Tobacco quit lines from 13 states participated in the study. Eligible participants were defined as quit line clients (ages 55-77 years) who reported a 30-plus pack-year smoking history and who spoke English. We excluded clients who reported a history of lung cancer. Tobacco quit line staff at the call centers asked new clients who met the age requirements of their interest in learning about lung cancer screening. Interested clients were then given a toll-free telephone number and an email address to contact the research team. Staff at the call centers also mailed recruitment materials to clients who met age requirements and who had contacted the tobacco quit line for smoking cessation services during the previous year. Mode of recruitment was tracked and included as a covariate in the outcome analyses. Research coordinators completed the eligibility assessment via telephone. Demographic data, contact information, and the baseline assessment were collected during the same telephone call.

### Randomization and Interventions

After completing the baseline assessment, clients within each state quit line were randomized to receive the PDA or standard educational material (EDU) using S-plus, version 8.04 (TIBCO Software Inc) statistical software to generate a randomization schedule with various block sizes. Participants were not blinded to intervention allocation. Study interviewers were blinded to participant allocation at the 3- and 6-month assessments, but not the 1-week follow-up because questions about the PDA were asked of participants in this group. Participants received the PDA intervention materials via mail in DVD format 1 week before the first follow-up assessment; they were also offered a weblink to the video (2 participants requested a weblink). Participants randomized to EDU were mailed a 2-page brochure about lung cancer screening. When needed, research coordinators assisted participants in finding a location where they could view the PDA such as a public library. Participants in both groups were encouraged to discuss screening with a health care clinician, but they were not given specific guidance on locating a screening facility.

#### Patient Decision Aid

The PDA was a 9.5-minute narrated video, *Lung Cancer Screening: Is It Right for Me?* (details of the development process have been previously described).^17,18^ In brief, the PDA was developed and refined iteratively with input from multiple stakeholders (eg, patient advocates, tobacco users, primary care clinicians, and tobacco cessation experts). The development process followed the standards of the International Patient Decision Aid Standards Collaboration,^21^ and the PDA met National Quality Forum certification criteria for PDAs.^22^ We updated our PDA to reflect the US Preventive Services Task Force 2014 recommendation^12^ and eligibility criteria for lung cancer screening from the CMS.^15^ The narrated PDA included information about (1) eligibility for lung cancer screening and a calculation of tobacco pack-year smoking history, (2) lung cancer epidemiology and risk factors, (3) a video of a patient in a CT scanner, (4) icon arrays to graphically depict the magnitude of mortality reduction, false-positive results, and harms from invasive diagnostic procedures, and (5) radiation exposure depicted within the context of other sources of radiation (eg, a screening mammogram). Smoking cessation was emphasized throughout the PDA.

#### Standard Educational Materials

The EDU material was a 2-page brochure from a lung cancer advocacy group and included structured questions a patient can ask a physician about lung cancer screening. The questions addressed (1) eligibility for screening, (2) the harms and benefits of screening, (3) what to expect from undergoing an LDCT scan, (4) the costs of screening, (5) how to interpret the LDCT results, (6) the importance of smoking cessation, and (7) where to find more information about lung cancer and screening. Benefits and harms were described but no probabilities of outcomes were included. Patient values related to the positive and negative features of lung cancer screening were not addressed (eTable 2 in Supplement 2).

### Outcomes

The primary outcomes were collected at the 1-week follow-up assessment and included preparation for decision-making and decisional conflict adapted for a decision about lung cancer screening. The Preparation for Decision Making Scale^23^ is a 10-item measure of the utility of the interventions in preparing the patient to communicate with a health care clinician about a screening decision. Two subscales from the Decisional Conflict Scale^24^ were also used: Informed Subscale, assessing perceived awareness of the advantages and disadvantages of lung cancer screening, and Values Clarity Subscale, an indicator of the perceived importance of the advantages and disadvantages of lung cancer screening in making a screening decision.

Secondary outcomes included knowledge of lung cancer screening^25^ at 1-week, 3-month, and 6-month follow-ups, intentions to be screened at the 1-week follow-up, and screening behaviors by the 6-month follow-up. Participants were asked if they scheduled or had a visit with their health care clinician to discuss lung cancer screening, and if they scheduled or had an LDCT scan since enrolling in the study. Acceptability of the PDA was assessed at the 1-week follow-up using questions adapted from the Ottawa Acceptability Measure.^26^

### Statistical Analysis

The primary analysis compared the 3 primary outcomes (Preparation for Decision Making Scale, Decisional Conflict Scale Informed subscale, and Decisional Conflict Scale Values Clarity subscale) between the PDA and EDU groups. We controlled overall type I error rate by adjusting for multiple comparisons using a Bonferroni correction and setting the threshold *P* = .017 (0.05/3).

Our accrual target was 500 participants, assuming 20% would be lost to follow-up by the 6-month follow-up. We estimated that the PDA group would have a mean Informed Subscale or Values Clarity Subscale score of 25 (lower decisional conflict) whereas the EDU group would have a mean Informed Subscale or Values Subscale of 30.^16,24,27,28^ A sample size of 190 in each arm provided 80% power to detect a difference in means of 5 using a 2-group *t* test with a 2-sided significance level of *P* = .017 assuming a common SD of 15 on the Informed Subscale or Values Subscale.^24^ For the Preparation for Decision Making Scale, a sample size of 190 in each arm provided 80% power for the study to detect an effect size of 0.332 using a 2-group *t* test with a 2-sided significance level of .017. We further used thresholds for determining the clinical significance of the primary outcome measures. For Preparation for Decision Making, we used a cut point of 75 or greater based on findings from the most recent Cochrane review^27^ to indicate participants being well prepared to make decisions after reviewing a PDA. Following the Decisional Conflict Scale manual, scores less than 25 were considered associated with implementing decisions about screening.^24^

Data analysis was conducted between May 5, 2017, and September 30, 2018. All analyses were conducted using SAS, version 9.4 (SAS Institute Inc) and S-plus, version 8.04 (TIBCO Software Inc) statistical software. Participant demographic and clinical characteristics at baseline were summarized descriptively.^29^ The 2-sided 2-group *t* tests were used to compare the differences of the 3 primary end points between the 2 study groups using intent-to-treat analysis. We tested for heterogeneity of treatment effects by examining the interaction between intervention group and race/ethnicity (black vs white, measured by participant reported race/ethnicity), level of education, and current vs former smoker at the time of study enrollment, using a linear regression model to examine whether or not the PDA had differential effects between participant subgroups. These analyses were conducted adjusting for multiple covariates including age, sex, race, educational level, insurance status, mode of administration and recruitment, and tobacco quit line call center.

Linear mixed-effects models for longitudinal measures^30,31^ were used to assess the change in the magnitude of lung cancer screening knowledge over time adjusting for the same covariates. Logistic regression analysis^32^ was used to assess the relationships between the primary outcome measures, screening intentions, screening behaviors and the intervention, and to assess the interactions between intervention and race/ethnicity with and without adjusting for the covariates. Analyses were conducted without imputation for missing data because missing data rates were low.

## Results

A total of 746 tobacco quit line clients were assessed for lung cancer screening eligibility (see Figure), of whom 230 were excluded and 516 randomized (259 to PDA and 257 to EDU). Of the 516 clients enrolled, 370 (71.7%) were younger than 65 years, 320 (62.0%) were female, 138 (26.7%) identified as black, 47 (9.1%) did not have health insurance, and 226 (43.8%) had a high school educational level or less. Follow-up rates were high: 235 of 259 PDA participants (90.7%) and 233 of 257 EDU participants (90.7%) completed the 1-week assessment, and 218 of 259 PDA participants (84.2%) and 225 of 257 EDU participants (87.5%) completed the 6-month assessment (eFigure in Supplement 2). Participants younger than 65 years were slightly more likely to complete the 1-week follow-up compared with participants 65 years and older (342 of 370 [92.4%] vs 126 of 146 [86.3%]; *P* = .03). No other differences were observed between participants who did and did not complete the follow-up assessments (eTable 3 in Supplement 2). Participant characteristics were similar between the 2 groups (Table 1).

**Figure.**
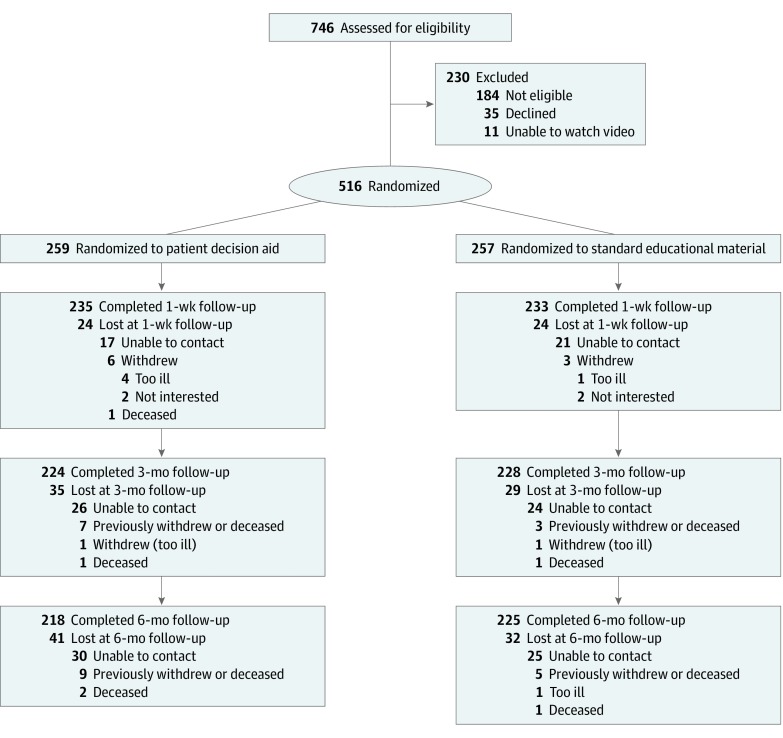
CONSORT Flow Diagram Participants (n = 20) missing 1-week follow-up assessments completed the 3-month follow-up; participants (n = 24) missing 3-month follow-up assessments completed the 6-month follow-up.

**Table 1.** Characteristics of the Trial Participants^a^

Characteristic	Patient Decision Aid Group (n = 259)	Standard Education Group (n = 257)	Total (N = 516)
Age, y			
≥65	69 (26.6)	77 (30.0)	146 (28.3)
<65	190 (73.4)	180 (70.0)	370 (71.7)
Sex			
Male	102 (39.4)	94 (36.6)	196 (38.0)
Female	157 (60.6)	163 (63.4)	320 (62.0)
Race/ethnicity^b^			
American Indian or Alaska Native	2 (0.8)	0	2 (0.4)
Asian	0	0	0
Black	62 (23.9)	76 (29.6)	138 (26.7)
Native Hawaiian or other Pacific Islander	0	1 (0.4)	1 (0.2)
Hispanic or Latino	7 (2.7)	1 (0.4)	8 (1.6)
White	185 (71.4)	177 (68.9)	362 (70.2)
Refused	0	1 (0.4)	1 (0.2)
More than 1 category	1 (0.4)	1 (0.4)	2 (0.4)
Other	2 (0.8)	0	2 (0.4)
Insurance			
Yes	239 (92.3)	230 (89.5)	469 (90.9)
No	20 (7.7)	27 (10.5)	47 (9.1)
Educational level			
Less than high school	41 (15.8)	36 (14.0)	77 (14.9)
Graduated high school or GED	72 (27.8)	77 (30.0)	149 (28.9)
Some college or trade school	107 (41.3)	105 (40.9)	212 (41.1)
Graduated college or more	39 (15.1)	39 (15.2)	78 (15.1)
Tobacco quitline call centers			
Alere, Seattle, Washington	21 (8.1)	19 (7.4)	40 (7.8)
Information & Quality Healthcare, Ridgeland, Mississippi	130 (50.2)	128 (49.8)	258 (50.0)
National Jewish Health, Denver, Colorado	40 (15.4)	43 (16.7)	83 (16.1)
Roswell Park, Buffalo, New York	68 (26.3)	67 (26.1)	135 (26.2)
Smoking history, median (IQR)			
Years smoked, No.	42.0 (40.0-49.0)	44.0 (40.0-50.0)	43.0 (40.0-50.0)
Cigarettes smoked per d, No.	20.0 (20.0-30.0)	20.0 (20.0-30.0)	20.0 (20.0-30.0)
Pack-year smoking history^c^	47.0 (40.0-63.0)	49.0 (40.0-63.8)	48.0 (40.0-63.0)

Abbreviation: GED, General Education Development; IQR, interquartile range.

^a^ Data are presented as number (percentage) of participants unless otherwise indicated.

^b^ Percentages may not sum to 100 because of rounding.

^c^ A pack-year is equivalent to smoking 1 pack of cigarettes (n = 20) a day for 1 year.

### Preparation for Decision-Making and Decisional Conflict

The PDA participants scored higher on the Preparation for Decision Making Scale than did EDU participants, indicating that they were better prepared to make a screening decision (Table 2). Similarly, PDA participants scored lower (better) on the Informed and Values Clarity subscales of the Decisional Conflict Scale than did EDU participants. Using cut points for clinical significance (eTable 4 in Supplement 2), 67.4% (153 of 227) of PDA participants were well prepared to make a screening decision compared with 48.2% (108 of 224) of EDU participants (odds ratio [OR], 2.31; 95% CI, 1.56-3.44; *P* < .001). For the Informed subscale of the Decisional Conflict Scale, 50.0% (117 of 234) of PDA participants compared with 28.3% (66 of 233) of EDU participants had low decisional conflict (OR, 2.56; 95% CI, 1.72-3.79; *P* < .001.) For the Value Clarity subscale, 68.0% (159 of 234) of PDA participants compared with 47.4% (110 of 232) of EDU participants had low decisional conflict (OR, 2.37; 95% CI, 1.6-3.51; *P* < .001). Tests for an interaction effect of intervention group and race/ethnicity (white or black) were not statistically significant for the primary outcomes.

**Table 2.** Differences in Preparation for Decision-Making and Decisional Conflict at the 1-Week Follow-up

Outcome Measure	Patient Decision Aid Group		Standard Education Group		Difference (95% CI)	*P* Value^a^
	No.	Score, Mean (95% CI)	No.	Score, Mean (95% CI)		
Preparation for Decision Making Scale	227	79.4 (77.1 to 81.7)	224	69.4 (66.4 to 72.4)	10.0 (6.3 to 13.8)	<.001
Decisional Conflict Scale						
Informed subscale^b^	234	27.1 (23.8 to 30.4)	233	42.1 (38.1 to 46.0)	−14.9 (−20.1 to −9.7)	<.001
Values Clarity subscale^b^	234	17.6 (14.2 to 21.0)	232	31.7 (27.4 to 35.9)	−14.1 (−19.5 to −8.7)	<.001

^a^ Tests were adjusted for age, sex, race/ethnicity, educational level, insurance status, quitline service provider, and recruitment method.

^b^ The Decisional Conflict Scales are scored from 0 to 100, with lower scores indicating lower decisional conflict about lung cancer screening. One decision aid participant and 1 standard education participant (Values Clarity subscale only) did not complete the Decisional Conflict Scale.

### Lung Cancer Screening Knowledge

Knowledge was significantly higher among the PDA participants than among the EDU participants at each follow-up assessment period (Table 3). Knowledge was highest at the 1-week follow-up for PDA participants, with a mean score of 57.5% (95% CI, 54.7%-60.3%) correct responses, but decreased over time. Among the PDA participants, knowledge was significantly lower at the 3-month (44.4%; 95% CI, 41.9%-47.0%) and 6-month (49.9%; 95% CI, 47.5%-52.3%) assessments compared with the 1-week assessment (*P* < .001). Among the EDU participants, compared with knowledge at the 1-week assessment (40.1%; 95% CI, 37.9%-42.3%), knowledge was significantly lower at the 3-month assessment (35.9%; 95% CI, 33.7%-38.1%) (*P* = .004) but did not differ from scores at the 6-month follow-up assessment (40.0%; 95% CI, 37.6%-42.4%) (*P* = .94).

**Table 3.** Correct Responses to Lung Cancer Screening Knowledge Measure by Study Group

Assessment Period	Patient Decision Aid Group		Standard Education Group		Difference (95% CI), %	*P* Value^a^
	No.	Correct Response, Mean (95% CI), %	No.	Correct Response, Mean (95% CI), %		
1 wk	235	57.5 (54.7-60.3)	233	40.1 (37.9-42.3)	17.4 (13.9-21.0)	<.001
3 mo	224	44.4 (41.9-47.0)	228	35.9 (33.7-38.1)	8.5 (5.1-11.9)	<.001
6 mo	218	49.9 (47.5-52.3)	225	40.0 (37.6-42.4)	9.9 (6.5-13.3)	<.001

^a^ Tests were adjusted for age, sex, race/ethnicity, educational level, insurance status, quitline service provider, and recruitment method.

### Screening Intentions and Behaviors

No group differences in intentions to be screened and screening behaviors were observed (Table 4). At the 1-week assessment, most participants intended to be screened within the next year (70.8% [165 of 233] who received the PDA, and 65.1% [151 of 232] who received EDU) and had scheduled a visit to discuss lung cancer screening with a health care clinician. By the 6-month follow-up, fewer PDA participants (70 of 237 [29.5%]) than EDU participants (89 of 238 [37.4%]) had scheduled LDCT, but the difference was not statistically significant. More than 80% of participants (PDA vs EDU: 85.1% [57 of 67] vs 80.0% [68 of 85]) who scheduled LDCT for lung cancer screening were screened by the 6-month follow-up.

**Table 4.** Screening Intentions and Behaviors by Study Group

Intent or Behavior	No./Total No. (%)^a^		Difference (95% CI), %	Odds Ratio (95% CI)^b^	*P* Value
	Patient Decision Aid Group	Standard Education Group			
Intent to be screened within 1 y at 1-wk assessment	165/233 (70.8)	151/232 (65.1)	5.7 (−2.7 to 14.2)	1.25 (0.83 to 1.89)	.29
Scheduled a visit with physician to discuss lung cancer screening by 6-mo follow-up	150/238 (63.0)	158/238 (66.4)	−3.4 (−11.9 to 5.2)	0.87 (0.59 to 1.28)	.47
Discussed lung cancer screening at visit with physician^c^	134/150 (89.3)	134/158 (84.8)	4.5 (−2.9 to 12.0)	1.43 (0.71 to 2.86)	.31
Scheduled CT for lung cancer screening by 6-mo follow-up	70/237 (29.5)	89/238 (37.4)	−7.9 (−16.2 to 1.0)	0.70 (0.47 to 1.03)	.07
Screened for lung cancer by 6-mo follow-up^d^	57/67 (85.1)	68/85 (80.0)	5.1 (−7.0 to 17.1)	1.27 (0.52 to 3.11)	.60

Abbreviation: CT, computed tomography.

^a^ Sample size varies owing to missing data.

^b^ Odds ratios and *P* values from logistic regression models were adjusted for the following covariates: age, sex, race/ethnicity, educational level, insurance status, quitline service provider, and recruitment method.

^c^ Among participants who scheduled a visit with a physician to discuss lung cancer screening.

^d^ Among participants who scheduled CT for lung cancer screening.

A significant interaction between intervention group and smoking status on scheduling LDCT was observed. Subgroup analyses indicated that among current smokers, the participants who were randomized to the PDA group (55 of 203 [27.1%]) were less likely to have scheduled LDCT by the 6-month follow-up than were participants randomized to the EDU group (81 of 209 [38.8%]; OR, 0.77; 95% CI, 0.62-0.94; *P* = .01). No other interaction effects involving intervention group and smoking status or level of education on the other screening behaviors were observed.

### Acceptability of the PDA

Only 10 of 228 participants (4.4%) felt that the PDA was too long, whereas 53 of 228 (23.2%) wanted more information. In addition, 198 of 227 participants (87.2%) indicated that the PDA included enough information to help a person make a decision about lung cancer screening.

## Discussion

In this randomized clinical trial of a PDA for lung cancer screening, smokers who received the PDA were better prepared to make a screening decision, reported lower decisional conflict, and had greater knowledge of the harms and benefits of screening compared with smokers who received EDU. The number of participants who intended to be screened within the next year was high and did not differ between the groups. Similarly, screening behaviors did not differ between the 2 groups, with nearly 2 in 3 participants having scheduled a visit with a health care clinician to discuss screening by the 6-month follow-up. These findings suggest that participants who received EDU were making decisions about lung cancer screening while feeling less prepared, being less clear about their values related to the harms and benefits, and having poorer knowledge of the harms and benefits than participants who received the PDA. Of note, the participants in this study were similar to clients served by tobacco quit lines nationally based on statistics reported by the North American Quitline Consortium^33^ and to participants in the National Lung Screening Trial based on age and pack-year smoking history.^34^

Approximately two-thirds (67.4%) of the participants in this study who received the PDA were well prepared to make screening decisions as a result of viewing the PDA compared with approximately one-half (48.2%) of the participants who received the EDU, a finding that is similar to or exceeds data from other studies of PDAs.^35,36,37,38^ Similarly, 50.0% to 68.0% of the PDA participants had low decisional conflict about their screening choice, which was approximately 20 percentage points better than that for participants who received the EDU. The magnitude of the differences between the 2 groups on the Decisional Conflict Scale Informed and Values Clarity subscales also exceeded the difference reported in the most recent Cochrane Systematic Review^27^ for PDAs delivered in preparation for a consultation with a health care clinician.

Participants who received the PDA had greater knowledge of lung cancer screening than did those who received the EDU, and these differences were maintained at each follow-up assessment. Of interest, approximately 1 in 4 participants wanted more information from the PDA; this highlights the importance of a conversation with a health care clinician to probe patients’ information needs and misconceptions related to lung cancer screening. Knowledge scores decreased by the 6-month follow-up assessment, suggesting that a refresher might be needed when the screening decision is reconsidered the following year for patients who have normal screening findings. Currently, CMS makes additional patient counseling and shared decision-making visits optional for subsequent annual screenings for lung cancer.^15^

Few studies have examined the effect of PDAs on lung cancer screening decision-making,^18,39,40,41^ and only 1 study, to our knowledge, used a comparison group.^42^ All studies^18,39,40,41^ were conducted in the United States and confirm our findings about increased knowledge and reduced decisional conflict^18,39^ as a result of receiving the PDA. The number of participants who intended to be screened for lung cancer was high in our study compared with other studies of primary care patients.^18,39,40,41^ Screening rates in this study were higher than national estimates^4^ but were lower than screening rates among referred patients in the pulmonary care setting,^40^ although few studies have examined this issue. Although we observed some differences in screening intentions and behaviors between PDA and EDU participants, future research with larger samples are needed to clarify the effect of PDAs on lung cancer screening behaviors. We would not necessarily expect a PDA to affect screening rates because enthusiasm for cancer screening in general is high.^43^ Our findings suggest that targeting smokers who are already motivated to quit smoking is an effective approach to increasing screening rates.

### Limitations

This study has limitations. Participants had to express an interest in lung cancer screening when asked by quit line call staff, and they had to contact the research team about participation. We were unable to explore the reasons why other smokers were not interested. For participants who had an appointment with a health care clinician to discuss lung cancer screening, we do not have information about the quality of the decision-making process or completion of screening after the 6-month follow-up. Screening behaviors were based on self-report.

## Conclusions

A PDA delivered to persons seeking services from tobacco quit lines improved the quality of lung cancer screening decisions compared with EDU. These improvements were consistent with recommendations of professional societies regarding smokers making informed decisions about lung cancer screening. The PDA had no differential effect on intentions to be screened or completion of screening. The PDA was meant to support but not replace a conversation with a health care clinician because there is a crucial need to improve the quality of these conversations.^44^ Disseminating the PDA through tobacco quit lines could reach a large number of potentially eligible smokers in the United States. Carefully addressing the role of tobacco quit lines in distributing PDA support for lung cancer screening, given variable quit line funding, is necessary for broader dissemination and greater effect of the intervention.

## Notes

Supplement 1. **Trial Protocol**

Supplement 2. **eTable 1.** IRB Approval Dates and Modifications to the Protocol **eTable 2.** Patient Decision Aid Compared With Standard Educational Materials According to NQF Screening Criteria **eTable 3.** Comparisons of Patients’ Characteristics by Status of Follow-up Assessments **eTable 4.** Comparison of Preparation for Decision Making and Decisional Conflict Scores Using Cut-points for Clinical Significance at 1-Week Follow-up **eFigure.** Participant Retention and Loss to Follow-up Across Assessment Periods

Supplement 3. **Data Sharing Statement**
