# Supplementary material for: Effect of a Patient Decision Aid on Lung Cancer Screening Decision-Making by Persons Who Smoke: A Randomized Clinical Trial
Source: JAMA Netw Open. 2020 Jan 31;3(1):e1920362. doi: 10.1001/jamanetworkopen.2019.20362 (PMC7042872; doi:10.1001/jamanetworkopen.2019.20362)
Supplement: Supplement 1. — Trial Protocol [file jamanetwopen-3-e1920362-s001.pdf]

THE UNIVERSITY OF TEXAS
MD Anderson ~~Cancer~~ Center

Protocol Page

Promoting Informed Decisions about Lung Cancer Screening: Randomized Trial 2014-0628

Core Protocol Information

**Short Title**	PCORI Lung Cancer Screening Trial
**Study Chair:**	Robert Volk
**Additional Contact:**	Marsha N. Richardson<br>Vincent F. Richards<br>Viola Leal
**Department:**	General Internal Medicine
**Phone:**	713-745-4516
**Unit:**	1465
**Full Title:**	Promoting Informed Decisions about Lung Cancer Screening: Randomized Trial
**Protocol Type:**	Standard Protocol
**Protocol Phase:**	N/A
**Version Status:**	IRB approved but not activated 10/24/2014
**Version:**	02
**Submitted by:**	Viola Leal--10/21/2014 3:59:29 PM
**OPR Action:**	Accepted by: David A. Kennedy -- 10/24/2014 3:26:43 PM

Which Committee will review this protocol?

(●) The Psychosocial Behavioral and Health Services Research Committee (PBHSRC)

Protocol Body

1.0 Rationale

Lung cancer is the second most common cancer in men and women, and the leading cause of cancer deaths in the US.[1] The American Cancer Society estimates that about 228,000 Americans will be diagnosed with lung cancer, and about 160,000 Americans will die of lung cancer this year.[1] Five-year survival rates are only about 16.6%, in part related to patients having advanced disease at the time of diagnosis.[2] Smoking is by far the most important risk factor for developing and dying from lung cancer. It is estimated that cigarette smoking causes about 90% of all lung cancers in the United States.[1, 3] Tobacco use is the #1 modifiable risk factor for death in the US, accounting for over 400,000 deaths annually. The morbidity associated with smoking is substantial. Tobacco use is associated with countless chronic health problems for smokers and others exposed to second hand smoke, including heart and lung disease.[4, 5]

In June 2011, the National Lung Screening Trial (NLST) published its primary findings. This landmark study found 20% fewer lung cancer deaths among current and former heavy smokers who were screened with low dose computed tomography (LDCT) compared to those who received standard chest x-rays.[6] Current estimates are that over 12,000 lung cancer deaths would be prevented each year if heavy smokers were screened annually.[7] The NLST findings were met with much fanfare in the media and were quickly followed by marketing campaigns promoting computed tomography (CT) scans for high-risk smokers. In fact, groups with an economic interest in testing had already begun offering CT screening for lung cancer after preliminary results of the NLST were released in November 2010.[8]

Yet, LDCT screening for lung cancer is not without risks, most notably, radiation exposure and a high false-positive rate leading to subsequent follow-up and testing with its own associated harms. For the individual, the benefit of being screened for lung cancer with LDCT must be weighed against the potential harms: receiving false results, radiation exposure, and the risk of over diagnosis.[6, 9] A recent systematic review of evidence regarding the benefits and harms of LDCT screening for lung cancer confirmed the results of the NLST while raising concerns about the potential harms of screening.[10] The rate of positive results in each round of screening was 20% while only 1% of patients were found to have lung cancer. Overall, patients who completed all three screens had a 40% chance of having at least 1 abnormal result, requiring additional surveillance and diagnostic workup.

The need for patient decision aids to support informed decision making for lung cancer screening with LDCT is growing dramatically. Evidence-based guidelines have been released endorsing LDCT scans for high risk smokers, while emphasizing the importance of making an informed decision about screening within the context of receiving smoking cessation services for people who continue to smoke.[10, 11] We know from our preliminary study (Protocol PA11-0626) that patients are interested in the topic, know very little of lung cancer screening and the potential harms and benefits, and want decision support tools.[12] In addition, insurers are increasingly covering

LDCT scans for lung cancer screening among patients meeting criteria consistent with the NLST. The US Preventive Services Task Force has issued a guideline endorsing annual screening with LDCT.[13] If followed by Medicaid and Medicare coverage, this would greatly increase the pool of high risk individuals able to receive the scans without copays. Thus, lung cancer screening is rapidly becoming a viable secondary preventive service for many heavy smokers, and there is a great need for quality, evidenced-based patient decision support to help patients make informed screening decisions.

The Patient-Centered Outcomes Research Institute (PCORI), an independent non-profit research organization established under the 2010 Patient Protection and Affordable Care Act,[14] has recognized this need. PCORI has awarded a contract to Dr. Robert Volk to update and evaluate a patient decision aid which promotes informed decision making about lung cancer screening with LDCT. This project, CER-1306-03385 “Promoting Informed Decisions about Lung Cancer Screening,” is in line with PCORI's mission to help people make informed healthcare decisions. The research plan was funded after peer review (attached as Appendix E).

2.0 Objectives

This protocol addresses the second phase of a larger project funded by the Patient-Centered Outcomes Research Institute to help heavy smokers make informed decisions about lung cancer screening with low-dose computed tomography (LDCT).

This is an educational research study. Patients of state-based tobacco quitlines will receive educational materials about lung cancer screening to help them understand the potential benefits and harms of screening. This is not a study to evaluate the outcomes of screening for lung cancer.

Our quitline partner for this study is Information & Quality Healthcare (IQH). IQH holds contracts to provide quitline services in the states of Mississippi and Alabama. They may be receiving new contracts for additional sites. Patients for this study will therefore be quitline patients from the states where IQH has contracts to provide services.

The aim of this phase is to compare outcomes for promoting informed screening decisions about lung cancer screening in a randomized trial of patients who smoke recruited through state-based smoking cessation quitlines, where patients will be randomly assigned to the updated patient decision aid or to standard educational materials on lung cancer screening.

We will test several hypotheses related to the use of the patient decision aid in promoting informed decisions about lung cancer screening. Specifically, compared to heavy smokers who receive standard educational materials which lack an emphasis on making an informed decision, heavy smokers who view the patient decision aid about lung cancer screening will report:

1. being more prepared to make a decision about lung cancer screening,
2. feeling more informed about the screening decision, and

3. being clearer about their values related to the tradeoffs between benefits and risks of lung cancer screening with LDCT.

We further hypothesize that heavy smokers who view the decision aid will be more knowledgeable about lung cancer screening than patients receiving the standard educational materials. We will also collect data about screening intentions and completion of screening for exploratory purposes. We make no a priori hypotheses about these latter outcomes because the literature on the impact of decision aids on cancer screening uptake is mixed.

3.0 Eligibility of Subjects

Inclusion Criteria:
1. Men and women 55 to 80 years of age.
2. Participants must speak English.
3. Current smoker or quit smoking within the past 15 years.
4. At least a 30 pack-year smoking history

Exclusion Criterion:
1. History of lung cancer.

Age Justification:
Current guidelines for lung cancer screening include people 55 to 80 years of age. These guidelines correspond to the recommendation of the U.S. Preventive Services Task Force. [13]

4.0 Research Plan and Methods

The design for this study is a randomized trial with quitline patients recruited through state-based quitlines (currently Mississippi and Alabama). Patients will be randomly assigned to the patient decision aid (200 patients) or standard educational information about lung cancer screening (200 patients). (Note that based on our estimates of attrition, up to 500 patients may be enrolled in the study to achieve a final sample of 400.) The design includes a pre-test assessment with a subscale of the knowledge measure developed for this study, an assessment at 1-week to collect data on the outcomes for the study and a 3-month follow-up to determine how well patients retain the information and track any use of screening services. A 6-month follow-up assessment will also be conducted on a subsample of the patients to track lung cancer screening rates via patient report.

Schema for the Randomized Trial

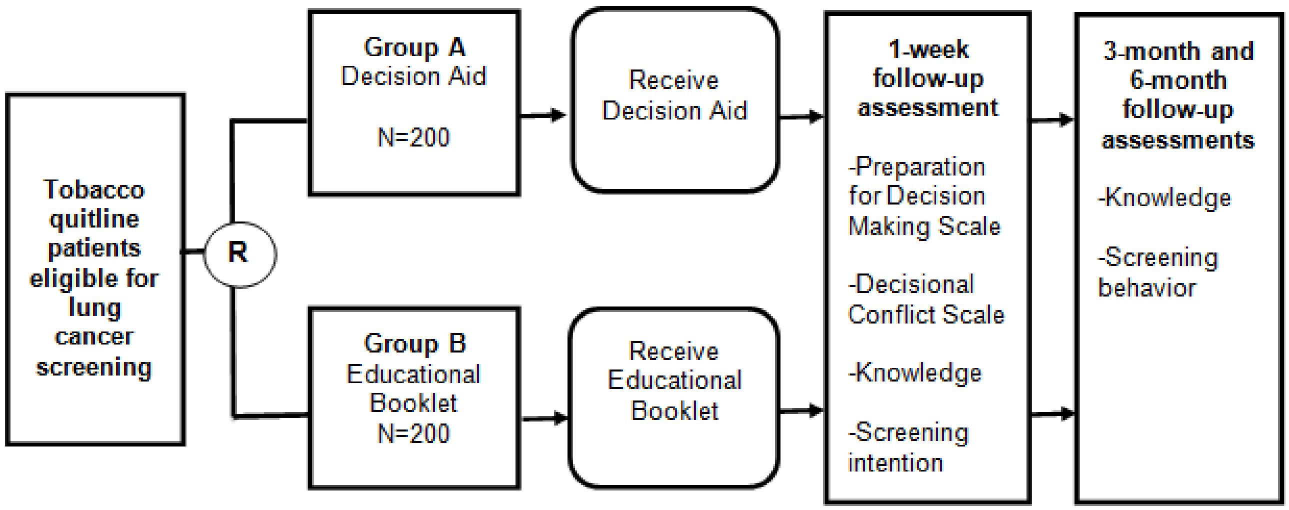

<u>Recruitment Strategies</u>
Subjects for this study will be patients referred by tobacco quitlines in states that have contracts with Information & Quality Healthcare (IQH) for provision of smoking cessation services. Thus, identification of patients for the study will be coordinated through IQH. Recruitment will occur over 24 months, starting in month 7 of year 1.

IQH will identify new referrals for smoking cessation services. This includes self-referrals and patients referred by primary care physicians and other health providers. We will also identify patients who are already registered with IQH. MD Anderson will not receive patient lists from IQH. Rather, IQH will send invitation materials directly to their patients.

If patients are between the ages of 55 and 80, IQH staff members will mail them invitation materials describing the research study. See the invitation letter in appendix F, the advertisement wording in appendix G, and the IRB advertisement approval form in appendix H. An invitation letter and advertisement will be placed in a QuitKit. The QuitKits include information about the different kinds of services provided (e.g., phone counseling, web-based information), how to access the services, and other resources available to patients to help them with quit smoking. In addition, patients who are already registered will be sent an email with a Portable Document Format file (PDF) of the invitation materials or be mailed the materials.

The recruitment materials that will be placed in the QuitKit will provide a toll free telephone number, an email address, and a postcard that patients can use to contact the research team about participation. When patients receive the materials and are interested in participating, they will contact MD Anderson staff who will assess their eligibility.

<u>Eligibility Assessment, Informed Consent, and Randomization</u>
Patients with an interest in the study will be given information to contact the research team about participation. Research staff will provide a description of the study using a recruitment script (appendix I) and assess the patients’ eligibility following the study

eligibility criteria using a screening form (appendix J). These forms will be kept for all individuals screened including screen-failures and used for aggregate reporting of eligibility. We propose to collect informed consent from patients via telephone. See Section 7 for details about the verbal consent process and request for waiver of written authorization for the study. Contact information (appendix I) and patient characteristics (see baseline survey in appendix K) will be collected at that time. Eligible, consented patients will then be allocated to receive the patient decision aid or standard educational materials using the procedure described under "Allocation Strategy." Randomization will be done within each state organization (i.e., pre-stratification by state quitline) to control for any systematic differences in clients served.

Patients will be mailed the study materials for review. Patients will be instructed to review the materials – patient decision aid or standard educational materials – over the following week. A link to the current patient decision aid and standard educational materials can be found in appendix L. Because the patient decision aid is a video, during the eligibility assessment we will ask patients if they have access to a DVD player or computer with a DVD drive for viewing the aid. We will also make the aid available for viewing on the Internet. If patients do not have access to a DVD player or have Internet access, we will facilitate their finding access locally (e.g., public libraries). In the data analysis phase, we will examine any differences in the study outcomes across the different modes for delivering the decision aid and adjust for these as needed.

1-week, 3-month, and 6-month Follow-up Assessments

When we enroll patients in the study we will schedule the 1-week follow-up (we allow up to 3 weeks after their scheduled follow-up to contact the patient). We will request contact information from patients at the time of enrollment. We will ask for names and contact information of additional persons who might be able to locate the patient if we cannot. During the 1-week follow-up assessment interview, we will administer the main outcome measures for the study (Preparation for Decision Making Scale, Decisional Conflict Scale), the secondary outcome measures (knowledge, screening intentions), and the measures of acceptability of the materials. See appendix M for the 1-week follow up survey. At the 3-month and 6-month follow-up assessments (we have up to three weeks after their scheduled follow-up date to contact the patients), we will repeat the knowledge measure and ask about any use of lung cancer screening services. See appendices N and O for the 3-month and 6-month follow-up surveys, respectively. If we are not able to complete the follow-up interviews by phone, we will mail a study questionnaire to the patient at the address they provide. A second mailing will be sent for patients who do not respond within 2 weeks of the initial mailing. Patients will receive $25 after completion of the 1-week and 3-month assessments in the form of gift cards mailed to them ($50 total). Patients asked to do the 6-month assessment will be mailed an additional $25 gift card when they complete it. Subject compensation will be tracked in a log (see appendix P).

5.0 Statistics and Justification of Sample Size

Justification of Sample Size

The primary analysis will be to compare three primary outcomes, i.e., Preparation for

Decision Making Scale, informed subscale (IS) and values subscale (VS) of Decisional Conflict Scale (DCS), between patients who were randomized to the patient decision aid arm (Arm 1) and those who were randomized to the standard educational materials (Arm 2). We specifically chose the IS and VS subscales because two of the International Patient Decision Aid Standards criteria for decision aids are that the aid helps individuals feel informed and clear about values. Since the three primary endpoints are equally of significance, to control overall type I error rate at a significant level of 0.05, we will compare each of the three primary outcomes at a significant level of 0.017 (0.05/3) using Bonferroni multiple comparison adjustment.

Based on results from other decision aid trials using the DCS, we estimate Arm 2 will have a mean DCS IS or VS score of 30 while Arm 1 will have a mean DCS IS or VS of 25.[15] A sample size of 190 in each arm will have 80% power to detect a difference in means of 5 using a two group t-test with a 0.017 two-sided significance level assuming a common standard deviation (SD) of 15 on IS or VS.[15] We calculated the effect size the study would be able to detect with the given sample size for preparation for decision making because we had limited preliminary data for this measure. A sample size of 190 in each arm will have 80% power for the study to detect an effect size of 0.332 using a two group t-test with a 0.017 two sided significance level.

As a secondary analysis, we will also compare the difference of knowledge scores between the two arms using two-sided two group t-test. We hypothesize that Aim 2 scores to be 50 (half the knowledge questions answered correctly) with a SD of 19.36.[12] We further expect, based on our pilot study, the knowledge scores for Aim 1 to have a SD of 15.76.[12] If we randomize 380 participants 1:1 to each of the two arms, we would have 88% power to detect a difference in knowledge scores of 5.75 (50 vs. 55.75) using a twosided two-sample t-test with unequal variances and alpha of 0.05. Therefore, the target final sample for the study will be 400 patients (200 per study arm). With an anticipated retention rate of 80% by the 3-month follow-up, 500 patients will be recruited for the study.

We will also estimate actual screening rate (ASR) for lung cancer within each arm and compare the ASRs between the two arms. We expect that approximately 300 participants will complete the 6-month follow-up assessment and provide information about completion of lung cancer screening (150/arm). A two group chi-square test with a 0.05 two-sided significance level will have 83% power to detect the difference between a ASR of 0.2 in Arm 2 and a ASR of 0.35 in Arm 1 (odds ratio of 2.154) when the sample size in each arm is 150.

Allocation Strategy

Participants will be randomized at 1:1 ratio into Group A or B. Randomization will be stratified by states and by various block sizes. The randomization will be carried out via the Clinical Trial Conduct (CTC) website (
https://biostatistics.mdanderson.org/ClinicalTrial
Conduct) which is housed on a secure server at MD Anderson and maintained by the MD Anderson Department of Biostatistics. Access to the website will be gained through

usernames and passwords provided by the MD Anderson Department of Biostatistics. Training on the use of the CTC will be provided by the biostatistical collaborator of the study before the study activation.

Analysis plan

Patients' demographic characteristics at baseline will be summarized using descriptive statistics such as mean, standard deviation, median, interquartile range (IQR), and frequency where appropriate. We will apply Student t-test/Wilcoxon test and Kruskal-Wallis test/analysis of variance (ANOVA) to compare continuous variables between arms, and the chi-square test or the Fisher's exact test to assess the differences of categorical variables between arms.[16]

As primary analysis, we will perform two-sided two group t-test to compare the differences of the three primary endpoints between the two arms. We will perform similar analysis on knowledge scores. For these efficacy endpoints, intend-to-treat analysis will be applied to the participants. We will look at the impact of race/ethnicity (African American vs. Caucasian) on the relationship between the outcomes and lung cancer screening materials using general linear regression. We will also assess the interaction between intervention and race/ethnicity using linear regression model to examine whether or not the decision aid has differential effects between African Americans and Caucasians by testing.

We will use two-sided two group t-test to compare the change of knowledge scores from baseline to post intervention between the two arms. Since the knowledge outcome will be assessed at week 1 and then 3-month and 6-month follow-ups, linear mixed effect models for longitudinal measures[17] will be employed to assess the change in the magnitude of the outcomes over time adjusting for multiple covariates including intervention indicator, age, gender, race, education level, insurance status, and other factors. We may also look at the interaction between intervention and time. Appropriate transformation of the measures will be used in the analyses to satisfy the normality assumption of linear mixed effect model. Mode of administration and completion of intervention/control materials (i.e., dose/exposure) will be tracked to account for their effects.

We will also estimate the lung cancer screening rate within each arm along with 95% confidence interval. Chi-square test will be used to assess the differences of the lung cancer screening rates between arms.

6.0 Data Security and Confidentiality

We are requesting a Data Safety Monitoring Board waiver on the grounds that this protocol presents no more than minimal risk to human subjects. The principal investigator (PI) will monitor the study. The research involves no procedures for which written consent is normally required outside of the research context. There is no medical or clinical intervention and no foreseeable risk of physical or economic harm. Possible psychological and social harms, which include worry about lung cancer and stigma associated with current or former smoking behavior, are no greater than the risks the subjects would encounter in their daily lives.

To protect confidentiality of the study participants, a unique study identification (ID) number will be assigned as an identifier. Subjects' participation in this study will be confidential and all data will be collected and coded in a confidential manner. The following data security procedures will be followed:

1. Collection of identifiers: Identifiers (name and contact information) will be collected but will be replaced by study ID numbers in the analytic file. The master database file that links the study ID to the participants' identifiers will be maintained on a secured server at MD Anderson in a password-protected file. The master database file will be accessible only to the principal investigator and those individuals on the delegation of authority log authorized to recruit and screen subjects for eligibility, consent, and collect data.
2. Data report: All data will be reported in aggregate format.
3. Training of personnel: Study personnel will be trained to maintain the confidentiality of personally-identifying information. All of the study personnel will complete the institution's human subjects protection training.
4. Data Storage: Patient information and relevant data will be stored on password-protected computers behind the institution firewall. Data will not be transferred to laptop computers that are removed from the institution. Paper records will be retained by the principal investigator (PI) in locked files.
5. Data Sharing: Study data will not be shared with any individuals or entities that are not involved in the study. Protected health information (PHI) will not be reused or disclosed to any other person or entity, or for other research.
6. Final Disposition of Study Records: These data will be used only for this research study. Identifiers including the key linking the study ID to the identifiers will be destroyed within 3 years of completion of the study. De-identified data will be retained indefinitely.

7.0 Informed Consent

After initial screening for eligibility, subjects will be consented. We propose to obtain informed consent from patients via telephone using a verbal consent script (attached as a specialized informed consent) and a verbal consent log (appendix Q). We are requesting a waiver of written authorization for the study (attached as a waiver of informed consent). We have used this approach in other educational research projects and patients find it highly acceptable. Patients will be given the option of participating in subsequent phases of this study or other research studies with an optional procedure for consent to recontact.

Since this protocol involves no more than minimal risk and does not involve the administration of drugs, radiotherapy, use of surgery, invasive procedures, or investigational devices, authority to obtain informed consent is delegated to individuals on the research staff who are not physicians. These individuals may include Professors, Instructors, Sr. Research Scientists, Postdoctoral Fellows, Program Coordinators, Clinical Studies Coordinators, Research Data Coordinators, Research Data Management Assistants, and Graduate Research Assistants who have completed Human Subjects Research Training.

8.0 References

1. American Cancer Society. Cancer facts & figures 2012 2012 [cited 2012 October 10]. Available from: http://www.cancer.org/acs/groups/content/@epidemiologysurveilance/documents/document/acspc-031941.pdf.
2. Howlader, N, Noone, AM, Krapcho, M, Garshell, J, Neyman, M, et al. SEER Stat Fact Sheets: Lung and Bronchus Bethesda, MD: National Cancer Institute; 2013 [cited 2013 August 13]. Available from: http://seer.cancer.gov/csr/1975_2010/, based on November 2012 SEER data submission, posted to the SEER web site, 2013.
3. Khan, N, Afaq, F, Mukhtar, H. Lifestyle as risk factor for cancer: Evidence from human studies. Cancer Letters. 2010;293(2):133-43.
4. Mokdad, AH, Marks, JS, Stroup, DF, Gerberding, JL. Actual causes of death in the United States, 2000. JAMA. 2004 Mar 10;291(10):1238-45. PubMed PMID: 15010446.
5. National Center for Chronic Disease Prevention and Health Promotion, Centers for Disease Control and Prevention. At A Glance: Tobacco Use 2011 [cited 2013 August 13]. Available from: http://www.cdc.gov/chronicdisease/resources/publications/aag/pdf/2011/Tobacco_AAG_2011_508.pdf.
6. Aberle, DR, Berg, CD, Black, WC, Church, TR, Fagerstrom, RM, et al. The National Lung Screening Trial: overview and study design. Radiology. 2011 Jan;258(1):243-53. PubMed PMID: 21045183. Pubmed Central PMCID: 3009383. Epub 2010/11/04.
7. Ma, J, Ward, EM, Smith, R, Jemal, A. Annual number of lung cancer deaths potentially avertable by screening in the United States. Cancer. 2013 Apr 1;119(7):1381-5. PubMed PMID: 23440730.

8. Sampson, D. American Cancer Society Pressroom Blog: And so it begins Atlanta, GA2012 [cited 2012 October 10]. Available from: http://acspressroom.wordpress.com/2010/11/09/and-so-it-begins/.
9. Aberle, DR, Adams, AM, Berg, CD, Black, WC, Clapp, JD, et al. Reduced lung-cancer mortality with low-dose computed tomographic screening. New England Journal of Medicine. 2011 Aug 4;365(5):395-409. PubMed PMID: 21714641. Epub 2011/07/01.
10. Bach, PB, Mirkin, JN, Oliver, TK, Azzoli, CG, Berry, DA, et al. Benefits and harms of CT screening for lung cancer: a systematic review. JAMA. 2012 Jun 13;307(22):2418-29. PubMed PMID: 22610500. Epub 2012/05/23.
11. Wender, R, Fontham, ET, Barrera, E, Jr., Colditz, GA, Church, TR, et al. American Cancer Society lung cancer screening guidelines. CA Cancer J Clin. 2013 Mar-Apr;63(2):107-17. PubMed PMID: 23315954. Pubmed Central PMCID: 3632634.
12. Volk, RJ, Linder, SK, Leal, V, Rabius, VA, Cinciripini, PM, et al. Patients' reactions to a decision aid about lung cancer screening with low-dose spiral computed tomography: An uncontrolled trial. Journal of Clinical Oncology, 2013 ASCO Annual Meeting Proceedings (Post-Meeting Edition). 2013;31(15_suppl):1564.
13. Moyer, VA. Screening for Lung Cancer: U.S. Preventive Services Task Force Recommendation Statement. Ann Intern Med. 2013 Dec 31. PubMed PMID: 24378917.
14. Patient-Centered Outcomes Research Institute. Mission and Vision 2014 [cited 2014 January 3]. Available from: http://www.pcori.org/about-us/mission-and-vision/.
15. Stacey, D, Bennett, CL, Barry, MJ, Col, NF, Eden, KB, et al. Decision aids for people facing health treatment or screening decisions. Cochrane Database Syst Rev. 2011 (10):CD001431. PubMed PMID: 21975733. Epub 2011/10/07.
16. Woolson, RF, Clarke, WR. Statistical methods for the analysis of biomedical data. 2nd ed. New York: Wiley; 2002.
17. Liang, KY. Longitudinal data analysis using generalized linear models. Biometrika. 1986;73:13-22.

THE UNIVERSITY OF TEXAS
MD Anderson Cancer Center

Protocol Page

Promoting Informed Decisions about Lung Cancer Screening: Randomized Trial 2014-0628

Core Protocol Information

**Short Title**	PCORI Lung Cancer Screening Trial
**Study Chair:**	Robert Volk
**Additional Contact:**	Marsha N. Richardson<br>Vincent F. Richards<br>Viola Leal
**Department:**	General Internal Medicine
**Phone:**	713-745-4516
**Unit:**	1465
**Full Title:**	Promoting Informed Decisions about Lung Cancer Screening: Randomized Trial
**Protocol Type:**	Standard Protocol
**Protocol Phase:**	N/A
**Version Status:**	Activated -- Closed to new patient entry as of 03/02/2017
**Version:**	10
**Submitted by:**	Vincent F. Richards--7/28/2016 2:35:26 PM
**OPR Action:**	Accepted by: Debbie D. Stroughter -- 8/12/2016 4:05:05 PM

Which Committee will review this protocol?

◉ The Psychosocial Behavioral and Health Services Research Committee (PBHSRC)

Protocol Body

1.0 Rationale

Lung cancer is the second most common cancer in men and women, and the leading cause of cancer deaths in the US.[1] The American Cancer Society estimates that about 228,000 Americans will be diagnosed with lung cancer, and about 160,000 Americans will die of lung cancer this year.[1] Five-year survival rates are only about 16.6%, in part related to patients having advanced disease at the time of diagnosis.[2] Smoking is by far the most important risk factor for developing and dying from lung cancer. It is estimated that cigarette smoking causes about 90% of all lung cancers in the United States.[1, 3] Tobacco use is the #1 modifiable risk factor for death in the US, accounting for over 400,000 deaths annually. The morbidity associated with smoking is substantial. Tobacco use is associated with countless chronic health problems for smokers and others exposed to second hand smoke, including heart and lung disease.[4, 5]

In June 2011, the National Lung Screening Trial (NLST) published its primary findings. This landmark study found 20% fewer lung cancer deaths among current and former heavy smokers who were screened with low dose computed tomography (LDCT) compared to those who received standard chest x-rays.[6] Current estimates are that over 12,000 lung cancer deaths would be prevented each year if heavy smokers were screened annually.[7] The NLST findings were met with much fanfare in the media and were quickly followed by marketing campaigns promoting computed tomography (CT) scans for high-risk smokers. In fact, groups with an economic interest in testing had already begun offering CT screening for lung cancer after preliminary results of the NLST were released in November 2010.[8]

Yet, LDCT screening for lung cancer is not without risks, most notably, radiation exposure and a high false-positive rate leading to subsequent follow-up and testing with its own associated harms. For the individual, the benefit of being screened for lung cancer with LDCT must be weighed against the potential harms: receiving false results, radiation exposure, and the risk of over diagnosis.[6, 9] A recent systematic review of evidence regarding the benefits and harms of LDCT screening for lung cancer confirmed the results of the NLST while raising concerns about the potential harms of screening.[10] The rate of positive results in each round of screening was 20% while only 1% of patients were found to have lung cancer. Overall, patients who completed all three screens had a 40% chance of having at least 1 abnormal result, requiring additional surveillance and diagnostic workup.

The need for patient decision aids to support informed decision making for lung cancer screening with LDCT is growing dramatically. Evidence-based guidelines have been released endorsing LDCT scans for high risk smokers, while emphasizing the importance of making an informed decision about screening within the context of receiving smoking cessation services for people who continue to smoke.[10, 11] We know from our preliminary study (Protocol PA11-0626) that patients are interested in the topic, know very little of lung cancer screening and the potential harms and benefits, and want decision support tools.[12] In addition, insurers are increasingly covering

LDCT scans for lung cancer screening among patients meeting criteria consistent with the NLST. The US Preventive Services Task Force has issued a guideline endorsing annual screening with LDCT.[13] If followed by Medicaid and Medicare coverage, this would greatly increase the pool of high risk individuals able to receive the scans without copays. Thus, lung cancer screening is rapidly becoming a viable secondary preventive service for many heavy smokers, and there is a great need for quality, evidenced-based patient decision support to help patients make informed screening decisions.

The Patient-Centered Outcomes Research Institute (PCORI), an independent non-profit research organization established under the 2010 Patient Protection and Affordable Care Act,[14] has recognized this need. PCORI has awarded a contract to Dr. Robert Volk to update and evaluate a patient decision aid which promotes informed decision making about lung cancer screening with LDCT. This project, CER-1306-03385 “Promoting Informed Decisions about Lung Cancer Screening,” is in line with PCORI's mission to help people make informed healthcare decisions. The research plan was funded after peer review (attached as Appendix E).

2.0 Objectives

This protocol addresses the second phase of a larger project funded by the Patient-Centered Outcomes Research Institute to help heavy smokers make informed decisions about lung cancer screening with low-dose computed tomography (LDCT).

This is an educational research study. Patients of state-based tobacco quitlines will receive educational materials about lung cancer screening to help them understand the potential benefits and harms of screening. This is not a study to evaluate the outcomes of screening for lung cancer.

Our quitline partners for this study are tobacco cessation counseling service providers who operate state-based quitlines as members of the North American Quitline Consortium. Patients for this study will therefore be quitline patients from participating states where these partners have contracts to provide services. This expands on our initial quitline partner, Information & Quality Healthcare (IQH).

The aim of this phase is to compare outcomes for promoting informed screening decisions about lung cancer screening in a randomized trial of patients who smoke recruited through state-based smoking cessation quitlines, where patients will be randomly assigned to the updated patient decision aid or to standard educational materials on lung cancer screening.

We will test several hypotheses related to the use of the patient decision aid in promoting informed decisions about lung cancer screening. Specifically, compared to heavy smokers who receive standard educational materials which lack an emphasis on making an informed decision, heavy smokers who view the patient decision aid about lung cancer screening will report:

1. being more prepared to make a decision about lung cancer screening,

2. feeling more informed about the screening decision, and
3. being clearer about their values related to the tradeoffs between benefits and risks of lung cancer screening with LDCT.

We further hypothesize that heavy smokers who view the decision aid will be more knowledgeable about lung cancer screening than patients receiving the standard educational materials. We will also collect data about screening intentions and completion of screening for exploratory purposes. We make no a priori hypotheses about these latter outcomes because the literature on the impact of decision aids on cancer screening uptake is mixed.

3.0 Eligibility of Subjects

Inclusion Criteria:
1. Men and women 55 to 77 years of age.
2. Participants must speak English.
3. Current smoker or quit smoking within the past 15 years.
4. At least a 30 pack-year smoking history

Exclusion Criterion:
1. History of lung cancer.

Age Justification:
The Centers for Medicare and Medicaid Services (CMS) coverage guidelines for lung cancer screening include people 55 to 77 years of age. [18]

4.0 Research Plan and Methods

The design for this study is a randomized trial with quitline patients recruited through state-based quitlines. Patients will be randomly assigned to the patient decision aid (200 patients) or standard educational information about lung cancer screening (200 patients). (Note that based on our estimates of attrition, up to 550 patients may be enrolled in the study to achieve a final sample of 400.) The design includes a pre-test assessment with a subscale of the knowledge measure developed for this study, an assessment at 1-week to collect data on the outcomes for the study and a 3-month follow-up to determine how well patients retain the information and track any use of screening services. A 6-month follow-up assessment will also be conducted on a subsample of the patients to track lung cancer screening rates via patient report.

Schema for the Randomized Trial

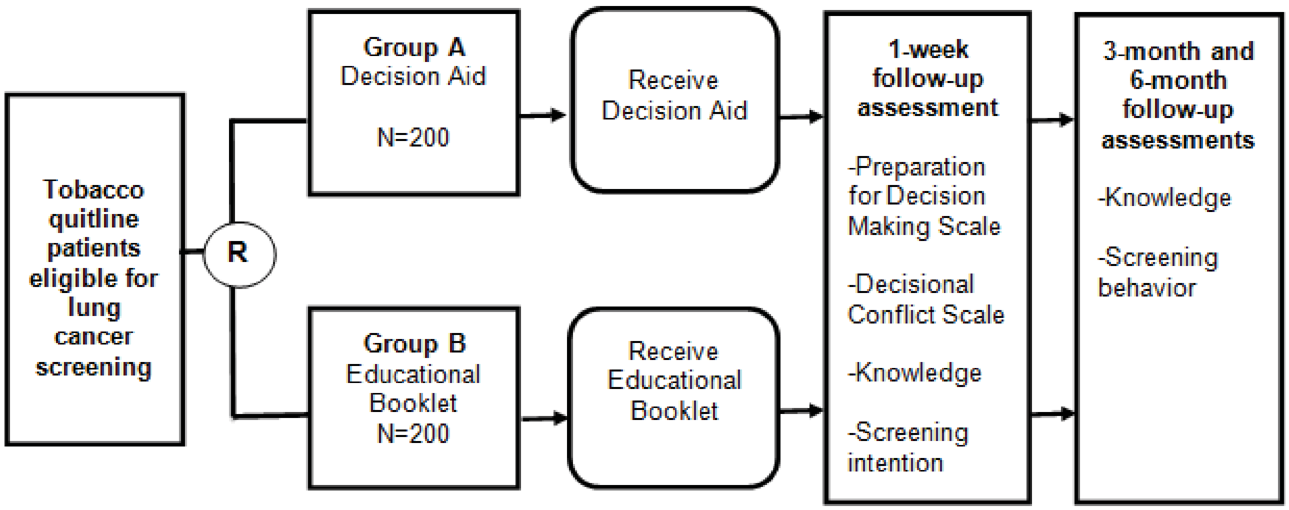

Recruitment Strategies

Subjects for this study will be patients referred by tobacco quitlines in states that have contracts with our quitline partners for provision of smoking cessation services. Thus, identification of patients for the study will be coordinated through the quitline service providers. Recruitment will occur over 24 months, starting in month 7 of year 1.

Our quitline partners will identify potential participants between the ages of 55 and 77. These may be new referrals for smoking cessation services (includes self-referrals and patients referred by primary care physicians and other health providers) or patients who are already registered with the quitline. MD Anderson will not receive patient lists from the quitline service providers. Rather, these quitline partners will send invitation materials directly to their patients.

Quitline staff members will mail potential participants invitation materials describing the research study or email them a Portable Document Format file (PDF) of the invitation materials. See the invitation letter in appendix F, the recruitment flyer in appendix G, and the IRB advertisement approval form in appendix H. The invitation letterhead and signature will be customized for each quitline partner.

An invitation letter and recruitment flyer will be placed in a QuitKit sent to new referrals at IQH. The QuitKits include information about the different kinds of services provided (e.g., phone counseling, web-based information), how to access the services, and other resources available to patients to help them with quit smoking. IQH staff members may also use the IQH script in Appendix R to inform new referrals about the study by phone.

The recruitment strategy may vary between quitlines. Like IQH, some quitlines may choose to mention the study by phone in addition to sending invitation materials. Other quitlines may choose to inform patients of our study solely by phone (see the quitline recruitment script in appendix Q).

The recruitment materials will provide a toll free telephone number and an email

address that patients can use to contact the research team about participation. When patients receive the materials and are interested in participating, they will contact MD Anderson staff who will assess their eligibility.

Eligibility Assessment, Informed Consent, and Randomization

Patients with an interest in the study will be given information to contact the research team about participation. Research staff will provide a description of the study using a recruitment script (appendix I) and assess the patients' eligibility following the study eligibility criteria using a screening form (appendix J). These forms will be kept for all individuals screened including screen-failures and used for aggregate reporting of eligibility. We propose to collect informed consent from patients via telephone. See Section 7 for details about the verbal consent process and request for waiver of written authorization for the study. Contact information (appendix I) and patient characteristics (see baseline survey in appendix K) will be collected at that time. Eligible, consented patients will then be allocated to receive the patient decision aid or standard educational materials using the procedure described under "Allocation Strategy." Randomization will be done within each state organization (i.e., pre-stratification by state quitline) to control for any systematic differences in clients served.

Patients will be mailed the study materials for review. Patients will be instructed to review the materials – patient decision aid or standard educational materials – over the following week. A link to the current patient decision aid and standard educational materials can be found in appendix L. Because the patient decision aid is a video, during the eligibility assessment we will ask patients if they have access to a DVD player or computer with a DVD drive for viewing the aid. We will also make the aid available for viewing on the Internet. If patients do not have access to a DVD player or have Internet access, we will facilitate their finding access locally (e.g., public libraries). In the data analysis phase, we will examine any differences in the study outcomes across the different modes for delivering the decision aid and adjust for these as needed.

1-week, 3-month, and 6-month Follow-up Assessments

When we enroll patients in the study we will schedule the 1-week follow-up (we allow up to 3 weeks after their scheduled follow-up to contact the patient). This is scheduled one week from the expected date of receipt of the intervention materials. If the patient did not receive the intervention materials, we re-send them and re-schedule the 1-week follow-up. The 3- and 6-month follow-ups are triggered by completion of prior follow-ups.

We will request contact information from patients at the time of enrollment. We will ask for names and contact information of additional persons who might be able to locate the patient if we cannot. During the 1-week follow-up assessment interview, we will administer the main outcome measures for the study (Preparation for Decision Making Scale, Decisional Conflict Scale), the secondary outcome measures (knowledge, screening intentions), and the measures of acceptability of the materials. See appendix M for the 1-week follow up survey. At the 3-month and 6-month follow-up assessments (we have up to three weeks after their scheduled follow-up date to contact the patients),

we will repeat the knowledge measure and ask about any use of lung cancer screening services. See appendices N and O for the 3-month and 6-month follow-up surveys, respectively.

If we are not able to complete the follow-up interviews by phone, we will mail a study questionnaire to the patient at the address they provide. Patients will receive $50 after completion of the 1-week assessment and $25 after completion of the 3-month assessment in the form of gift cards mailed to them ($75 total). Patients asked to do the 6-month assessment will be mailed an additional $25 gift card when they complete it. Subject compensation will be tracked in a log (see appendix P).

5.0 Statistics and Justification of Sample Size

Justification of Sample Size

The primary analysis will be to compare three primary outcomes, i.e., Preparation for Decision Making Scale, informed subscale (IS) and values subscale (VS) of Decisional Conflict Scale (DCS), between patients who were randomized to the patient decision aid arm (Arm 1) and those who were randomized to the standard educational materials (Arm 2). We specifically chose the IS and VS subscales because two of the International Patient Decision Aid Standards criteria for decision aids are that the aid helps individuals feel informed and clear about values. Since the three primary endpoints are equally of significance, to control overall type I error rate at a significant level of 0.05, we will compare each of the three primary outcomes at a significant level of 0.017 (0.05/3) using Bonferroni multiple comparison adjustment.

Based on results from other decision aid trials using the DCS, we estimate Arm 2 will have a mean DCS IS or VS score of 30 while Arm 1 will have a mean DCS IS or VS of 25.[15] A sample size of 190 in each arm will have 80% power to detect a difference in means of 5 using a two group t-test with a 0.017 two-sided significance level assuming a common standard deviation (SD) of 15 on IS or VS.[15] We calculated the effect size the study would be able to detect with the given sample size for preparation for decision making because we had limited preliminary data for this measure. A sample size of 190 in each arm will have 80% power for the study to detect an effect size of 0.332 using a two group t-test with a 0.017 two sided significance level.

As a secondary analysis, we will also compare the difference of knowledge scores between the two arms using two-sided two group t-test. We hypothesize that Aim 2 scores to be 50 (half the knowledge questions answered correctly) with a SD of 19.36.[12] We further expect, based on our pilot study, the knowledge scores for Aim 1 to have a SD of 15.76.[12] If we randomize 380 participants 1:1 to each of the two arms, we would have 88% power to detect a difference in knowledge scores of 5.75 (50 vs. 55.75) using a twosided two-sample t-test with unequal variances and alpha of 0.05. Therefore, the target final sample for the study will be 400 patients (200 per study arm). With an anticipated retention rate of 80% by the 3-month follow-up, 550 patients will be recruited for the study.

We will also estimate actual screening rate (ASR) for lung cancer within each arm and

compare the ASRs between the two arms. We expect that approximately 300 participants will complete the 6-month follow-up assessment and provide information about completion of lung cancer screening (150/arm). A two group chi-square test with a 0.05 two-sided significance level will have 83% power to detect the difference between a ASR of 0.2 in Arm 2 and a ASR of 0.35 in Arm 1 (odds ratio of 2.154) when the sample size in each arm is 150.

Allocation Strategy

Participants will be randomized at 1:1 ratio into Group A or B. Randomization will be stratified by states and by various block sizes. The randomization will be carried out via the Clinical Trial Conduct (CTC) website (
https://biostatistics.mdanderson.org/ClinicalTrial
Conduct) which is housed on a secure server at MD Anderson and maintained by the MD Anderson Department of Biostatistics. Access to the website will be gained through usernames and passwords provided by the MD Anderson Department of Biostatistics. Training on the use of the CTC will be provided by the biostatistical collaborator of the study before the study activation.

Analysis plan

Patients' demographic characteristics at baseline will be summarized using descriptive statistics such as mean, standard deviation, median, interquartile range (IQR), and frequency where appropriate. We will apply Student t-test/Wilcoxon test and Kruskal-Wallis test/analysis of variance (ANOVA) to compare continuous variables between arms, and the chi-square test or the Fisher's exact test to assess the differences of categorical variables between arms.[16]

As primary analysis, we will perform two-sided two group t-test to compare the differences of the three primary endpoints between the two arms. We will perform similar analysis on knowledge scores. For these efficacy endpoints, intend-to-treat analysis will be applied to the participants. We will look at the impact of race/ethnicity (African American vs. Caucasian) on the relationship between the outcomes and lung cancer screening materials using general linear regression. We will also assess the interaction between intervention and race/ethnicity using linear regression model to examine whether or not the decision aid has differential effects between African Americans and Caucasians by testing.

We will use two-sided two group t-test to compare the change of knowledge scores from baseline to post intervention between the two arms. Since the knowledge outcome will be assessed at week 1 and then 3-month and 6-month follow-ups, linear mixed effect models for longitudinal measures[17] will be employed to assess the change in the magnitude of the outcomes over time adjusting for multiple covariates including intervention indicator, age, gender, race, education level, insurance status, and other factors. We may also look at the interaction between intervention and time. Appropriate transformation of the measures will be used in the analyses to satisfy the normality assumption of linear mixed effect model. Mode of administration and completion of intervention/control materials (i.e., dose/exposure) will be tracked to account for their

effects.

We will also estimate the lung cancer screening rate within each arm along with 95% confidence interval. Chi-square test will be used to assess the differences of the lung cancer screening rates between arms.

6.0 Data Security and Confidentiality

We are requesting a Data Safety Monitoring Board waiver on the grounds that this protocol presents no more than minimal risk to human subjects. The principal investigator (PI) will monitor the study. The research involves no procedures for which written consent is normally required outside of the research context. There is no medical or clinical intervention and no foreseeable risk of physical or economic harm. Possible psychological and social harms, which include worry about lung cancer and stigma associated with current or former smoking behavior, are no greater than the risks the subjects would encounter in their daily lives.

To protect confidentiality of the study participants, a unique study identification (ID) number will be assigned as an identifier. Subjects' participation in this study will be confidential and all data will be collected and coded in a confidential manner. The following data security procedures will be followed:

1. Collection of identifiers: Identifiers (name and contact information) will be collected but will be replaced by study ID numbers in the analytic file. The master database file that links the study ID to the participants' identifiers will be maintained on a secured server at MD Anderson in a password-protected file. The master database file will be accessible only to the principal investigator and those individuals on the delegation of authority log authorized to recruit and screen subjects for eligibility, consent, and collect data.
2. Data report: All data will be reported in aggregate format.
3. Training of personnel: Study personnel will be trained to maintain the confidentiality of personally-identifying information. All of the study personnel will complete the institution's human subjects protection training.
4. Data Storage: Data will be stored on password-protected computers behind the institution firewall and in REDCap. Data on password-protected computers will include these identifiers: name, address, telephone numbers, and email addresses. The only identifiers stored in REDCap will be ZIP codes. Data will not be transferred to laptop computers that are removed from the institution. Paper records will be retained by the principal investigator (PI) in locked files.
5. Data Sharing: Study data will not be shared with any individuals or entities that are not involved in the study. Protected health information (PHI) will not be reused or disclosed to any other person or entity, or for other research.
6. Final Disposition of Study Records: These data will be used only for this research study. Identifiers on password-protected computers including the key linking the study ID to the identifiers will be destroyed within 3 years of completion of the study. De-identified data on password-protected computers will be retained indefinitely. Data in REDCap will be archived rather than destroyed, including ZIP codes.

REDCap

Study data will be managed using REDCap (Research Electronic Data Capture) electronic data capture tools hosted at MD Anderson.[19] REDCap (www.project-redcap.org) is a secure, web-based application with controlled access designed to support data capture for research studies, providing: 1) an intuitive interface for validated data entry; 2) audit trails for tracking data manipulation and export procedures; 3) automated export procedures for seamless downloads to common statistical packages; and 4) procedures for importing data from external sources. In the case of multi-center studies REDCap uses Data Access Groups (DAGs) to ensure that personnel at each institution are blinded to the data from other institutions. REDCap (https://redcap.mdanderson.org) is hosted on a secure server by MD Anderson Cancer Center's Department of Research Information Systems & Technology Services.

REDCap has undergone a Governance Risk & Compliance Assessment (05/14/14) by MD Anderson's Information Security Office and found to be compliant with HIPAA, Texas Administrative Codes 202-203, University of Texas Policy 165, federal regulations outlined in 21CFR Part 11, and UTMDACC Institutional Policy #ADM0335. Those having access to the data file include the study PI and research team personnel. Users are authenticated against MDACC's Active Directory system. External collaborators are given access to projects once approved by the project sponsor. The application is accessed through Secure Socket Layer (SSL). All protected health information (PHI) will be removed from the data when it is exported from REDCap for analysis. All dates for a given patient will be shifted by a randomly generated number between 0 and 364, thus preserving the distance between dates. Dates for each patient will be shifted by a different randomly generated number. Following publication study data will be archived in REDCap.

7.0 Informed Consent

After initial screening for eligibility, subjects will be consented. We propose to obtain informed consent from patients via telephone using a verbal consent script (attached as a specialized informed consent). The CORe registration list will serve as a verbal consent log for accrual tracking. We are requesting a waiver of written authorization for the study (attached as a waiver of informed consent). We have used this approach in other educational research projects and patients find it highly acceptable. Patients will be given the option of participating in subsequent phases of this study or other research studies with an optional procedure for consent to recontact.

Since this protocol involves no more than minimal risk and does not involve the administration of drugs, radiotherapy, use of surgery, invasive procedures, or investigational devices, authority to obtain informed consent is delegated to individuals on the research staff who are not physicians. These individuals may include Professors, Instructors, Sr. Research Scientists, Postdoctoral Fellows, Program Coordinators, Clinical Studies Coordinators, Research Data Coordinators, Research Data Management Assistants, and Graduate Research Assistants who have completed Human Subjects Research Training.

8.0 References

1. American Cancer Society. Cancer facts & figures 2012 2012 [cited 2012 October 10]. Available from: http://www.cancer.org/acs/groups/content/@epidemiologysurveilance/documents/document/acspc-031941.pdf.
2. Howlader, N, Noone, AM, Krapcho, M, Garshell, J, Neyman, M, et al. SEER Stat Fact Sheets: Lung and Bronchus Bethesda, MD: National Cancer Institute; 2013 [cited 2013 August 13]. Available from: http://seer.cancer.gov/csr/1975_2010/, based on November 2012 SEER data submission, posted to the SEER web site, 2013.
3. Khan, N, Afaq, F, Mukhtar, H. Lifestyle as risk factor for cancer: Evidence from human studies. Cancer Letters. 2010;293(2):133-43.
4. Mokdad, AH, Marks, JS, Stroup, DF, Gerberding, JL. Actual causes of death in the United States, 2000. JAMA. 2004 Mar 10;291(10):1238-45. PubMed PMID: 15010446.
5. National Center for Chronic Disease Prevention and Health Promotion, Centers for Disease Control and Prevention. At A Glance: Tobacco Use 2011 [cited 2013 August 13]. Available from: http://www.cdc.gov/chronicdisease/resources/publications/aag/pdf/2011/Tobacco_AAG_2011_508.pdf.
6. Aberle, DR, Berg, CD, Black, WC, Church, TR, Fagerstrom, RM, et al. The National Lung Screening Trial: overview and study design. Radiology. 2011 Jan;258(1):243-53. PubMed PMID: 21045183. Pubmed Central PMCID: 3009383. Epub 2010/11/04.
7. Ma, J, Ward, EM, Smith, R, Jemal, A. Annual number of lung cancer deaths potentially avertable by screening in the United States. Cancer. 2013 Apr 1;119(7):1381-5. PubMed PMID: 23440730.
8. Sampson, D. American Cancer Society Pressroom Blog: And so it begins Atlanta, GA2012 [cited 2012 October 10]. Available from: http://acspressroom.wordpress.com/2010/11/09/and-so-it-begins/.
9. Aberle, DR, Adams, AM, Berg, CD, Black, WC, Clapp, JD, et al. Reduced lung-cancer mortality with low-dose computed tomographic screening. New England Journal of Medicine. 2011 Aug 4;365(5):395-409. PubMed PMID: 21714641. Epub 2011/07/01.
10. Bach, PB, Mirkin, JN, Oliver, TK, Azzoli, CG, Berry, DA, et al. Benefits and harms of CT screening for lung cancer: a systematic review. JAMA. 2012 Jun 13;307(22):2418-29. PubMed PMID: 22610500. Epub 2012/05/23.
11. Wender, R, Fontham, ET, Barrera, E, Jr., Colditz, GA, Church, TR, et al. American Cancer Society lung cancer screening guidelines. CA Cancer J Clin. 2013 Mar-Apr;63(2):107-17. PubMed PMID: 23315954. Pubmed Central PMCID: 3632634.
12. Volk, RJ, Linder, SK, Leal, V, Rabius, VA, Cinciripini, PM, et al. Patients' reactions to a decision aid about lung cancer screening with low-dose spiral computed tomography: An uncontrolled trial. Journal of Clinical Oncology, 2013 ASCO Annual Meeting Proceedings (Post-Meeting Edition). 2013;31(15_suppl):1564.
13. Moyer, VA. Screening for Lung Cancer: U.S. Preventive Services Task Force

Recommendation Statement. Ann Intern Med. 2013 Dec 31. PubMed PMID: 24378917.

14. Patient-Centered Outcomes Research Institute. Mission and Vision 2014 [cited 2014 January 3]. Available from: http://www.pcori.org/about-us/mission-and-vision/.
15. Stacey, D, Bennett, CL, Barry, MJ, Col, NF, Eden, KB, et al. Decision aids for people facing health treatment or screening decisions. Cochrane Database Syst Rev. 2011 (10):CD001431. PubMed PMID: 21975733. Epub 2011/10/07.
16. Woolson, RF, Clarke, WR. Statistical methods for the analysis of biomedical data. 2nd ed. New York: Wiley; 2002.
17. Liang, KY. Longitudinal data analysis using generalized linear models. Biometrika. 1986;73:13-22.
18. Services, C. f. M. M. (2014). "Decision Memo for Screening for Lung Cancer with Low Dose Computed Tomography (LDCT) (CAG-00439N)." Retrieved 02/06/2015, 2015, from http://www.cms.gov/medicare-coverage-database/details/nca-decision-memo.aspx?NCAId=274.
19. Harris, PA, Taylor, R, Thielke, R, Payne, J, Gonzalez, N, Conde, JG. Research electronic data capture (REDCap) - A metadata-driven methodology and workflow process for providing translational research informatics support. J Biomed Inform, 2009. 42(2):377-81.
