# Supplementary material for: Effect of a Patient Decision Aid on Lung Cancer Screening Decision-Making by Persons Who Smoke: A Randomized Clinical Trial
Source: JAMA Netw Open. 2020 Jan 31;3(1):e1920362. doi: 10.1001/jamanetworkopen.2019.20362 (PMC7042872; doi:10.1001/jamanetworkopen.2019.20362)
Supplement: Supplement 2. — eTable 1. IRB Approval Dates and Modifications to the Protocol eTable 2. Patient Decision Aid Compared With Standard Educational Materials According to NQF Screening Criteria eTable 3. Comparisons of Patients’ Characteristics by Status of Follow-up Assessments eTable 4. Comparison of Preparation for Decision Making and Decisional Conflict Scores Using Cut-points for Clinical Significance at 1-Week Follow-up eFigure. Participant Retention and Loss to Follow-up Across Assessment Periods [file jamanetwopen-3-e1920362-s002.pdf]

Supplementary Online Content

Volk RJ. Lowenstein LM, Leal VB, et al. Effect of a patient decision aid on lung cancer screening decision-making by persons who smoke: a randomized clinical trial. *JAMA Netw Open*. 2020;3(1):e1920362. doi:10.1001/jamanetworkopen.2019.20362

eTable 1. IRB Approval Dates and Modifications to the Protocol

eTable 2. Patient Decision Aid Compared With Standard Educational Materials According to NQF Screening Criteria

eTable 3. Comparisons of Patients' Characteristics by Status of Follow-up Assessments

eTable 4. Comparison of Preparation for Decision Making and Decisional Conflict Scores Using Cut-points for Clinical Significance at 1-Week Follow-up

eFigure. Participant Retention and Loss to Follow-up Across Assessment Periods

This supplementary material has been provided by the authors to give readers additional information about their work.

© 2020 Volk RJ, et al. *JAMA Network Open.*

eTable 1. IRB Approval Dates and Modifications to the Protocol

Date Approved	Description of Modification
3/26/2015	Protocol approved and activated
3/27/2015	Shortened baseline knowledge questionnaire; added educational materials as appendix
5/1/2015	Updated follow-up questionnaires as appendices
9/1/2015	Dropped one state quitline and added another because of change in service provider contract; clarified recruitment strategies to allow for mailing of recruitment materials to previous quitline clients; modified incentive payment schedule for follow-up assessments; added baseline knowledge questions to follow-up questionnaires
3/4/2016	Clarified timing of follow-up questionnaires based on receipt of study interventions; moved study databases to REDCap per institutional policy
3/2/2017	Closed protocol to new subject entry

Activation indicates study is approved to begin subject accrual.

© 2020 Volk RJ, et al. *JAMA Network Open.*

eTable 2. Patient Decision Aid Compared With Standard Educational Materials According to NQF Screening Criteria

NQF Screening Criteria	Patient Decision Aid	Standard Educational Materials
1. The patient decision aid describes the health condition or problem for which a decision is required	Describes what lung cancer is, describes mortality (lung cancer specific and all cause), incidence, diagnosis, and risk factors	States lung cancer death as the health problem
2. The patient decision aid identifies the target user	Uses USPSTF eligibility criteria for LCS	Uses NLST eligibility criteria for LCS
3. The patient decision aid explicitly states the decision under consideration	Whether or not to be screened	Whether or not to be screened
4. The patient decision aid describes the options available for the decision, including non-treatment when appropriate	Screening with low dose CT vs. no screening	Screening with low dose CT vs. no screening
5. The patient decision aid describes the positive features of each option	States that LCS reduces the lung cancer specific risk of dying by 16-20%; leads to fewer deaths from all causes; can find other health problems that may be treated earlier	States that screening for lung cancer may save your life
6. The patient decision aid describes the negatives features of each option	Exposure to radiation; false positives with further testing (e.g. higher radiation scans, biopsy) and associated complications; overdiagnosis	Radiation risk; need for additional tests and procedures
7a. The patient decision aid clarifies patient values for outcomes of options by:<br>-Asking patients to consider or rate which positive and negative features matter most to them; and/or	Asks viewer to think about the reasons they would choose to be screened and the reasons they would choose not to be screened	Not addressed
7b. The patient decision aid clarifies patient values for outcomes of options by:<br>-Describing the features of options to help patients imagine the physical and/or social and/or psychological effects	Describes benefits (reduces lung cancer deaths and deaths from all causes), and the harms (false positives, additional testing, radiation exposure, overdiagnosis)	Not addressed

NQF, National Quality Forum; USPSTF, United States Preventive Services Task Force; NLST, National Lung Screening Trial; LCS, lung cancer screening; CT, computed tomography

© 2020 Volk RJ, et al. *JAMA Network Open.*

eTable 3. Comparisons of Patients' Characteristics by Status of Follow-up Assessments

	Completed 1-week follow-up?					Completed 3-month follow-up?					Completed 6-month follow-up?				
	Yes		No		*P* value	Yes		No		*P* value	Yes		No		*P* value
	n	(%)	n	(%)		n	(%)	n	(%)		n	(%)	n	(%)	
**Age**															
65+ years	126	86.3	20	13.7	.03	128	87.7	18	12.3	.97	130	89.0	16	11.0	.19
Under 65 years	342	92.4	28	7.6		324	87.6	46	12.4		313	84.6	57	15.4	
**Gender**															
Male	178	90.8	18	9.2	.94	174	88.8	22	11.2	.53	171	87.2	25	12.8	.48
Female	290	90.6	30	9.4		278	86.9	42	13.1		272	85.0	48	15.0	
**Race**[a]															
White	327	90.1	36	9.9		311	85.7	52	14.3		306	84.3	57	15.7	
Black or African American	131	94.9	7	5.1	.95	128	92.8	10	7.2	.35	125	90.6	13	9.4	.25
Other	10	66.7	5	33.3		13	86.7	2	13.3		12	80.0	3	20.0	
**Insurance**															
Yes	428	91.3	41	8.7	.17	412	87.8	57	12.2	.59	403	85.9	66	14.1	.88
No	40	85.1	7	14.9		40	85.1	7	14.9		40	85.1	7	14.9	
**Education**															
Less than high school	69	89.6	8	10.4	.59	71	92.2	6	7.8	.22	67	87.0	10	13.0	.64
Graduated high school/GED	134	89.9	15	10.1		127	85.2	22	14.8		128	85.9	21	14.1	
Some college/ trade school	191	90.1	21	9.9		182	85.8	30	14.2		178	84.0	34	16.0	
Graduated college or more	74	94.9	4	5.1		72	92.3	6	7.7		70	89.7	8	10.3	

© 2020 Volk RJ, et al. *JAMA Network Open.*

eTable 3. Comparisons of Patients' Characteristics by Status of Follow-up Assessments, Continued.

	Completed 1-week follow-up?					Completed 3-month follow-up?					Completed 6-month follow-up?				
	Yes		No		*P* value	Yes		No		*P* value	Yes		No		*P* value
	n	(%)	n	(%)		n	(%)	n	(%)		n	(%)	n	(%)	
**Quitline Call Centers**															
Alere	37	92.5	3	7.5	.11	37	92.5	3	7.5	.15	34	85.0	6	15.0	.35
Information & Quality Healthcare	230	89.1	28	10.9		220	85.3	38	14.7		215	83.3	43	16.7	
National Jewish Health	81	97.6	2	2.4		78	94	5	6.0		73	88.0	10	12.0	
Roswell Park	120	88.9	15	11.1		117	86.7	18	13.3		121	89.6	14	10.4	
**Method of recruitment**															
Callers to quitline	196	93.3	14	6.7	.09	191	91.0	19	9.0	.06	180	85.7	30	14.3	.94
Previous clients	272	88.9	34	11.1		261	85.3	45	14.7		263	85.9	43	14.1	

[a] Statistics tests only include white and African American categories because few patients endorsed a different racial category.

© 2020 Volk RJ, et al. *JAMA Network Open.*

eTable 4. Comparison of Preparation for Decision Making and Decisional Conflict Scores Using Cut-points for Clinical Significance at 1-Week Follow-up

Scales[a]	PDA: Decision Aid Group		EDU: Standard Education Group		Difference (95% CI), *percentage points*		
	n/N	%	n/N	%		Odds Ratio (95% CI)[b]	*P* value
**Preparation for Decision Making Scale** (well prepared, scores of 75 or greater)	153/227	67.4	108/224	48.2	19.2 (10.2 to 28.1)	2.31 (1.56, 3.44)	< .001
**Decisional Conflict Scale: Informed Subscale**							
Low Decisional Conflict (scores less than 25)	117/234	50.0	66/233	28.3	21.7 (13.0 to 30.3)	2.56 (1.72, 3.79)	< .001
High Decisional Conflict (scores greater than 37.5)	51/234	21.8	103/233	44.2	-22.4 (-30.7 to -14.1)	0.32 (0.21, 0.49)	< .001
**Decisional Conflict Scale: Values Clarity Subscale**							
Low Decisional Conflict (scores less than 25)	159/234	68.0	110/232	47.4	20.5 (11.8 to 29.3)	2.37 (1.6, 3.51)	< .001
High Decisional Conflict (scores greater than 37.5)	39/234	16.7	78/232	33.6	-17.0 (-24.7 to -9.2)	0.42 (0.26, 0.66)	< .001

Sample size varies due to missing data.
[a] The Preparation for Decision Making Scale is scored on a 0 to 100 scale, with higher scores indicated better preparation for making a decision after using the PDA or EDU materials. Scores of 75 of greater were considered clinically significant based on previous research. The Decisional Conflict Scales are scored on 0 to 100 scales, with lower scores indicating lower decisional conflict about lung cancer screening. Scores under 25 are associated with implementing decisions; scores greater than 37.5 are associated with delaying decisions or feeling unsure about implementation.
[b] Odds ratios and P values from logistic regression models, adjusted for the following covariates: age, gender, race, education level, insurance status, quitline service provider, and recruitment method.

© 2020 Volk RJ, et al. *JAMA Network Open.*

eFigure. Participant Retention and Loss to Follow-up Across Assessment Periods

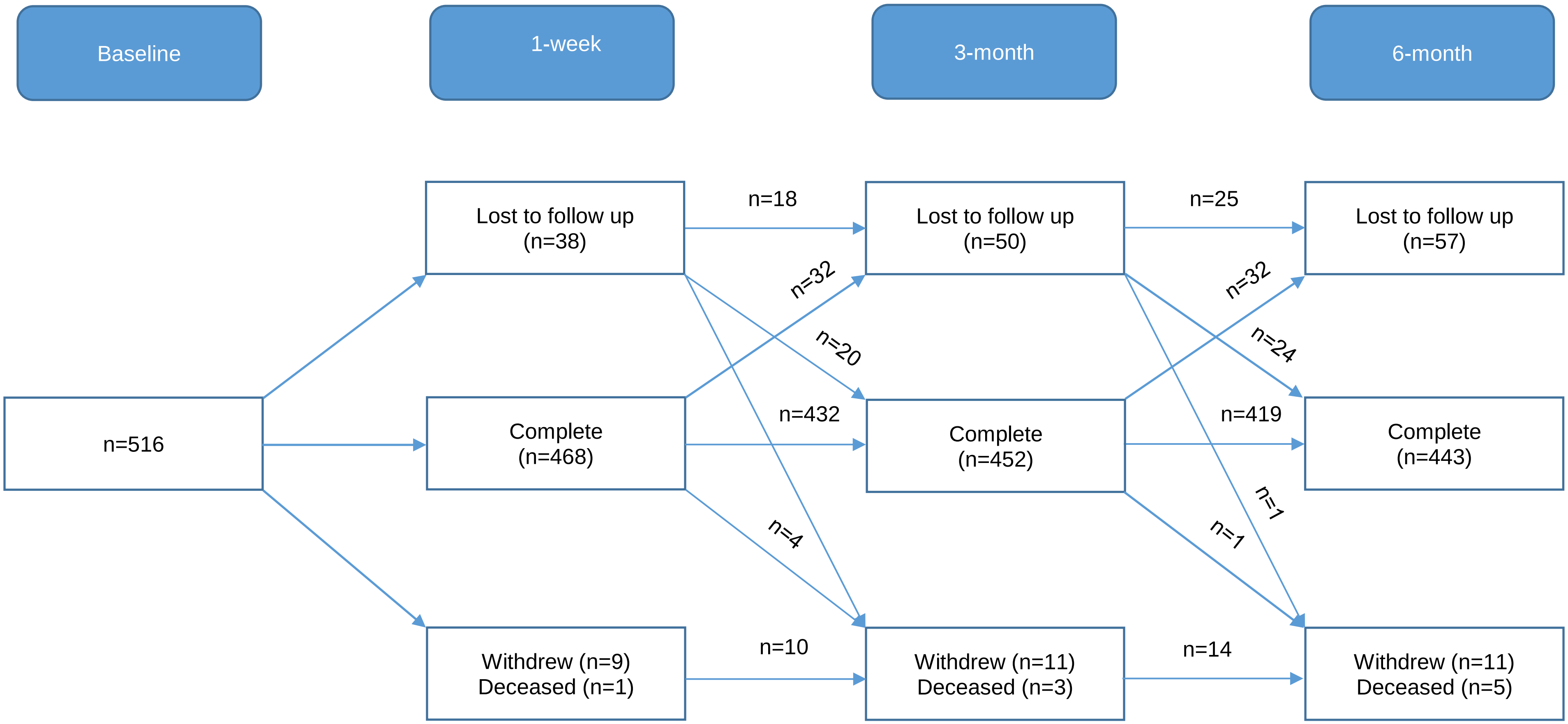

© 2020 Volk RJ, et al. *JAMA Network Open.*
