# Supplementary material for: Effect of a Patient Decision Aid on Lung Cancer Screening Decision-Making by Persons Who Smoke: A Randomized Clinical Trial
Source: JAMA Netw Open. 2020 Jan 31;3(1):e1920362. doi: 10.1001/jamanetworkopen.2019.20362 (PMC7042872; doi:10.1001/jamanetworkopen.2019.20362)
Supplement: Supplement 3. — Data Sharing Statement [file jamanetwopen-3-e1920362-s003.pdf]

Data Sharing Statement

Volk. Effect of a Patient Decision Aid on Lung Cancer Screening Decision-Making by Persons Who Smoke. *JAMA Netw Open*. Published January 31, 2020. 10.1001/jamanetworkopen.2019.20362

Data
Data available: Yes
Data types: Deidentified participant data, Data dictionary
How to access data: Available to approved persons through an agreement with the authors (email: bvolk@mdanderson.org)
When available: beginning date: 01-01-2021

Supporting Documents
Document types: Informed consent form
How to access documents: Requests for the informed consent document can be made to the corresponding author (bvolk@mdanderson.org).
When available: With publication

Additional Information
Who can access the data: Available to approved persons through an agreement with the authors (email: bvolk@mdanderson.org).
Types of analyses: Available to approved persons through an agreement with the authors (email: bvolk@mdanderson.org).
Mechanisms of data availability: Available to approved persons through an agreement with the authors (email: bvolk@mdanderson.org).
